# Sex Differences in Neuromuscular Fatigability of the Knee Extensors Post-Stroke

**DOI:** 10.3390/brainsci7010008

**Published:** 2017-01-12

**Authors:** Meghan Kirking, Reivian Berrios Barillas, Philip Andrew Nelson, Sandra Kay Hunter, Allison Hyngstrom

**Affiliations:** 1 Department of Physical Therapy, Marquette University, Milwaukee, WI 53201, USA; meghan.kirking@gmail.com (M.K.); sandra.hunter@marquette.edu (S.K.H.); 2 Department of Occupational Therapy, Concordia University, Mequon, WI 53097, USA; Reivian.BerriosBarillas@cuw.edu; 3 Department of Physical Medicine and Rehabilitation, Medical College of Wisconsin, Milwaukee, WI 53201, USA; panelson@mcw.edu

**Keywords:** fatigability, muscle fatigue, knee extensors, sex differences, chronic stroke, gender

## Abstract

Background and Purpose: Despite the implications of optimizing strength training post-stroke, little is known about the differences in fatigability between men and women with chronic stroke. The purpose of this study was to determine the sex differences in knee extensor muscle fatigability and potential mechanisms in individuals with stroke. Methods: Eighteen participants (10 men, eight women) with chronic stroke (≥6 months) and 23 (12 men, 11 women) nonstroke controls participated in the study. Participants performed an intermittent isometric contraction task (6 s contraction, 3 s rest) at 30% of maximal voluntary contraction (MVC) torque until failure to maintain the target torque. Electromyography was used to determine muscle activation and contractile properties were assessed with electrical stimulation of the quadriceps muscles. Results: Individuals with stroke had a briefer task duration (greater fatigability) than nonstroke individuals (24.1 ± 17 min vs. 34.9 ± 16 min). Men were more fatigable than women for both nonstroke controls and individuals with stroke (17.9 ± 9 min vs. 41.6 ± 15 min). Individuals with stroke had less fatigue-related changes in muscle contractile properties and women with stroke differed in their muscle activation strategy during the fatiguing contractions. Conclusions: Men and women fatigue differently post-stroke and this may be due to the way they neurally activate muscle groups.

## 1. Introduction

Women and men with chronic stroke have long-term motor impairments which contribute to disability [1]. For example, lower limb paresis is strongly associated with walking deficits [2,3,4]. Current stroke rehabilitation paradigms may not be optimized because it is assumed that the muscle of men and women fatigue similarly in response to fatiguing exercise and ultimately strengthen similarly after repeated bouts of fatiguing exercise followed by adequate rest. This assumption however, has not been demonstrated in the stroke literature and in healthy adults, there are significant sex differences in fatigability of skeletal muscle [5]. Understanding the impact of sex on neuromuscular fatigability will assist in developing training protocols that can be individualized and tailored to the sex of the individual in order to optimize strength training and motor recovery.

In both men and women with stroke, recent evidence demonstrates that the limb muscles are more fatigable than healthy controls [6,7]. Fatigability can be quantified as the acute, exercise-induced reduction in maximal force or power of a muscle or the time to failure (task duration) of a submaximal intensity task [8]. This greater neuromuscular fatigability with stroke compared with healthy controls was observed as a larger reduction in maximal dynamic voluntary contractions [6] and a reduced time to failure of sustained isometric contractions [7]. Accordingly, we have shown that fatigability of the hip flexor muscles is associated with walking speed in people with stroke [6], so that people with stroke who have greater hip flexion fatigability have larger decrements in walking performance [9]. These results are consistent with clinical studies showing limited walking endurance [10] and low quality of movement [11,12,13,14] in people with stroke.

As with healthy controls, the mechanisms of fatigability post-stroke are both neural and muscular in origin, but in contrast to healthy controls, neural mechanisms contribute more to fatigability in stroke survivors than healthy controls [15]. Ultimately, failure to adequately activate muscle during contractions during fatiguing exercise that aims to increase strength, potentially minimizes overload of the muscle and the strength gains during rehabilitation.

Whether or not sex differences exist in fatigability post-stroke has not been systemically examined and if it exists may have implications for prescription of fatiguing exercise during rehabilitation for the sexes. Typically, studies evaluating sex-related differences post-stroke focus on risk factors, prediction of stroke, and the acute medical management [1,16,17,18,19,20,21]. In other neurological populations, such as multiple sclerosis, there is evidence of sex differences in neuromuscular fatigue [22]. In healthy people, men are usually stronger than women, but they are more fatigable than women for maximal and submaximal contractions when assessed at the same relative intensity [5,22]. This sex difference in fatigability is attributed primarily to muscular mechanisms [23,24,25] leading to a greater accumulation of metabolites in the exercising muscle that is thought to be responsible for larger decreases in voluntary activation in men than women for the lower limb muscles [26,27]. Whether neural mechanisms that exacerbate the sex difference in fatigability of the lower limb in healthy people also has similar affects in people post-stroke is not known.

The purpose of this study was to quantify sex-related differences in knee extensor fatigability for a sub-maximal isometric contraction of the knee extensor muscles in people with chronic stroke and healthy controls. We hypothesized that individuals with stroke would be more fatigable than controls and that sex differences would persist. In addition to primary dependent measurements of torque generation and task duration, contractile properties and global surface electromyography (EMG) were measured to provide mechanistic insight into fatigability post-stroke in men and women.

## 2. Materials and Methods

### 2.1. Subjects

All activities in this study were approved by the Institutional Review Board at the Medical College of Wisconsin (PRO15398). All participants gave written informed consent prior to study participation. Eighteen participants (10 men and eight women) with chronic stroke (≥6 months, see Table 1 for stroke subject details) and 23 (12 men, 55 y.o. ± 8 and 11 women, 64.2 y.o. ± 9) community dwelling neurologically intact subjects were recruited. Stroke subject inclusion criteria: (1) history of a single, unilateral stroke and (2) the ability to ambulate at least 30 feet with or without an assistive device. Stroke subject exclusion criteria: (1) history of multiple strokes; (2) could not walk >10 feet without physical support or (3) inability to follow 2–3 step commands. All subjects were excluded from this study if they had low back pain, history of knee soreness and/or surgery, or risk factors associated with exercise.

### 2.2. Experimental Setup and Data Collection

Custom written LabVIEW programs were used here to collect the torque and EMG data (sampled at 1 kHz). Using a PCI DAQ (National Instruments), the torque and EMG data were then low-pass filtered at 500 Hz and sampled at 1 kHz at the time of data collection.

### 2.3. Torque Measurements

Each subject was seated on a System 3 Dynamometer (Biodex Medical Systems, Shirley, NY, USA) instrumented with a JR3 E-series 6-axis load cell (JR3, Inc., Woodland, CA, USA) in order to measure knee torque. The knees and hips were positioned at 90° of flexion.

### 2.4. Electromyography Measurements

Electromyography (EMG) was recorded with disposable adhesive gel bipolar electrodes (Vermed^®^, Illmehau, Germany) placed on the muscle bellies of the vastus medialis (VM), and rectus femoris (RF). An AMT-8 Octopus (Bortec Electronic, Inc., Calgary, AB, Canada) amplified the electrode leads 1,000–10,000 times.

### 2.5. Resting Twitch Measurements

Resting twitch measurements were made in a subset of the subjects (control: five women and six men; and stroke: six women and six men). A brief constant-current stimulator (Digitimer DS7AH, Welwyn Garden City, UK) delivered a rectangular pulse of 100 µs duration with maximum amplitude of 400 V, which was used to stimulate the rectus femoris muscle. The stimulation intensity (usually 200 mA to 500 mA) was set at 20% above the level required to produce a maximal resting twitch amplitude that caused a supramaximal stimulation.

### 2.6. Experimental Protocol

Subjects first performed 3–5 isometric knee extensor maximal voluntary contractions (MVC). Subjects were verbally instructed to “kick as hard as you can and as fast as you can” and to “relax as fast as you can”. MVCs efforts were repeated until there was less than a 5% difference in torque between two subsequent MVCs. At least 1 min rest was given between subsequent MVCs. The fatigue protocol consisted of a series of five, 6-s isometric submaximal knee extensor contractions at 30% of the subject’s MVC. An MVC was performed at the end of the five contractions. This set of contractions (five submaximal MVCs followed by one MVC) was repeated until one of the three criteria for fatigue was met: (1) if during one of the submaximal MVCs the subject’s torque production dropped below 5% of the target torque for more than half of the contraction; (2) if during one of the submaximal MVCs the subject’s torque production dropped below 5% of the target torque three or more times; or (3) if the fatigue protocol lasted 60 min.

### 2.7. Data Processing

All EMG and torque data were processed using custom Matlab (The Mathworks, Natick, MA, USA) programs. Initial processing included: (1) calibration of the 6-axis load cell to output *Z*-axis (coronal plane) torque; (2) low-pass filtering (15 Hz) of the *Z*-axis torque using a fourth-order zero-phase Butterworth filter (*filtfilt* command in Matlab); (3) correction for the ‘baseline’ knee torque produced by weight of the leg in the brace; and (4) band-pass filtering (10–499 Hz) of EMG using a fourth-order Butterworth filter. Both torque and EMG data were also band-reject filtered (59–61 Hz) to remove 60 Hz line voltage.

The peak MVC (all subjects) and resting twitch amplitude (subset indicated above in Resting Twitch sub-section) were calculated before and after the fatigue protocol and used to determine the percent declines in each variable, respectively. The peak rates of relaxation and contraction were calculated from each MVC and then each variable averaged across the trials before and after the fatigue protocol. The root mean square (RMS) of the EMG signal was calculated during a 3 s epoch ending at relaxation time using a 100-ms sliding window. This was done for the first five and last five contractions during the fatigue protocol. Percent change of the mean RMS between the first and last five contractions was then calculated for VM and RF for each subject group.

### 2.8. Statistical Analysis

Data are reported as means ± stdev. A student’s *t*-test was used to detect differences in clinical measures of function (Lower Extremity Fugl–Meyer Assessment, Berg Balance assessment, and self-selected walking speed) between men and women with stroke (α = 0.05). Separate two way ANOVAs were used to detect differences between groups (stroke and control), sex (male and female) and interactions (sex × group) for the following variables: baseline MVCs, percent decline in MVC, task duration, percent decline in resting twitch amplitude, and percent change in EMG. Separate mixed model repeated measures ANOVAs were used to detect between and within group differences for peak rates of contraction and relaxation during MVCs. To detect significant interaction effects, repeated *t*-tests were used (Bonferroni correction, α = 0.016). Partial eta squared values are reported for estimates of effect size. The coefficient of determination (*r*^2^) was calculated to determine relationships between task duration and baseline knee extensor MVCs, resting twitch amplitudes, Fugl–Meyer Scores, Berg Balance Scores, and self-selected walking speed. Because there were no significant differences detected between men and women with respect to their clinical scores, scores were grouped together for the correlations with fatigue metrics. Only significant correlations are reported.

## 3. Results

### 3.1. Baseline Measurements

Clinical assessment scores of the stroke subjects indicated motor deficits compared with normative values, but scores were similar between the men and women with stroke for the Berg Balance Assessment, Lower Extremity Fug–-Meyer, and self-selected walking speed (Table 1).

Baseline MVC measurements were as follows: (1) men controls = 179.1 ± 20 Nm; (2) women controls = 82.0 ± 40 Nm; (3) men with stroke = 84.3 ± 28 Nm; and (4) women with stroke = 41 ± 12 Nm. There were main effects of group and sex for the baseline MVC in that controls had greater MVCs than stroke (*p* < 0.001, η_p_^2^ = 0.613) and men were stronger than women (*p* < 0.001, η_p_^2^ = 0.629). There was an interaction effect between sex × group (*p* = 0.006, η_p_^2^ = 0.203), where on average there was a 50% difference between men and women with stroke and 45% difference between men and women without stroke. The mean MVC of men with stroke was larger than women with stroke (*p* < 0.01), but less than men without stroke (*p* < 0.01). Additionally, men with stroke had comparable MVC strength as compared with women without stroke (*p* = 0.88).

Baseline resting twitch amplitude data in a subset of participants (11 control and 12 with stroke) were as follows: (1) men without stroke = 45.5 ± 4 Nm; (2) women without stroke = 23.0 ± 6 Nm; (3) men with stroke 41 ± 20 Nm; and (4) women with stroke = 16.3 ± 6 Nm. As a main effect, men had a larger twitch amplitude than women (*p* ≤ 0.01, η_p_^2^ = 0.593). There was no effect of group (*p* = 0.15) and no interaction effect of sex × group (*p* = 0.86).

### 3.2. Fatigability

**Figure 1:**
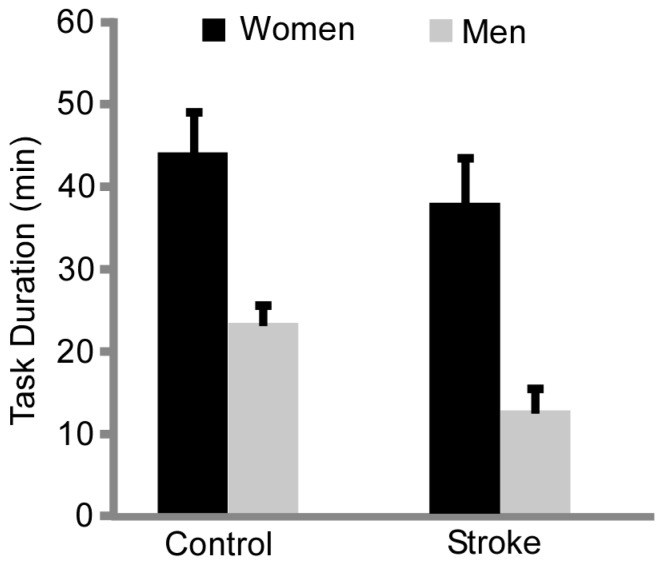
Task Duration. The stroke subjects had a shorter task duration compared with the control subjects (group effect, *p* = 0.01). Men with stroke had a shorter task duration compared to the control men and women, and women with stroke (*p* ≤ 0.001).

Stroke participants had a shorter task duration compared with the control group (24.1 ± 17 min vs. 34.9 ± 16 min, group effect, *p* = 0.045, η_p_^2^ = 0.113) and men had a shorter task duration than women (Figure 1, 17.9 ± 9 min vs. 41.6 ± 15 min, sex effect, *p* < 0.001, η_p_^2^ = 0.492). There were no interactions between sex × group (*p* = 0.57).

There was a negative correlation between task duration and baseline MVC torque for the men controls (*r*^2^ = 0.52, *p* = 0.01) such that those with greater strength had a briefer task duration. For all the other groups, there was no association including the women controls (*r*^2^ = 0.02, *p* = 0.65), men with stroke (*r*^2^ = 0.01, *p* = 0.72) or women with stroke (*r*^2^ < 0.01, *p* =0.98).

Despite the differences in task duration, there were no differences in the percent reduction in knee extensor MVC at termination of the fatiguing task between sex or group (*p* ≥ 0.92) and no interaction (*p* = 0.74). The reductions for women controls were 51.2% ± 11%, men controls 57.5% ± 14%, women stroke 44.4% ± 10% and men stroke = 53.5% ±18%.

Rates of contraction and relaxation at the start and end of the MVC respectively, also differed across groups and with fatigue. Peak contraction rates were faster before versus after the fatigue protocol (time effect: pre-fatigue = 184.36 ± 142 Nm/s vs. 150.2 ± 136 Nm/s, *p* = 0.03, η_p_^2^ = 0.131). Men had a faster rate of contraction (209 ± 85 Nm/s) compared with women (101.66 ± 88 Nm/s, *p* = 0.001, η_p_^2^ = 0.288). Controls (224 ± 91 Nm/s) were faster than stroke (87 ± 91 Nm/s, *p* < 0.001, η_p_^2^ = 0.397). There was an interaction effect between sex × group (*p* = 0.023, η_p_^2^ = 0.152). Men without stroke had the fastest rates of contraction *p* < 0.01 compared with men with stroke and both female groups

Peak relaxation rates were faster before than after the fatigue protocol (time effect: pre-fatigue = −174.3 ± 174 Nm/s vs. −123.4 ± 100 Nm/s, *p* = 0.004, η_p_^2^ = 0.229). Men had faster peak rates of relaxation (−184.1 ± 89 Nm/s) as compared to women (−92.1 ± 91 Nm/s, *p* = 0.005, η_p_^2^ = 0.220). Controls (−210 ± 93 Nm/s) were faster than stroke (−65 ± 91 Nm/s, *p* < 0.001, η_p_^2^ = 0.413). There was an interaction effect between sex × group (*p* = 0.008, η_p_^2^ = 0.196). Men without stroke had the fastest peak rate of relaxation compared to men with stroke and females from both groups (*p* < 0.01).

**Figure 2:**
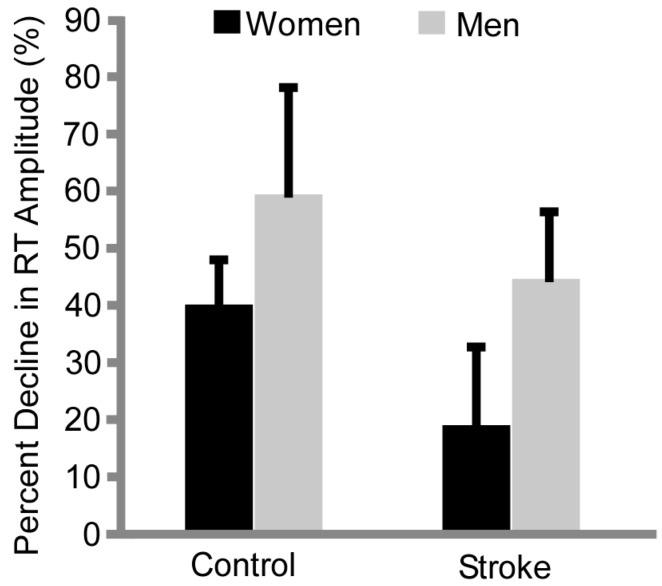
Percent Decline in Resting Twitch (RT) Amplitude. The control subjects had a greater decline (% from baseline) in resting twitch amplitude than the stroke subjects (*p* = 0.006) and men had greater declines in resting twitch amplitude than women (*p* = 0.001).

In the subset of participants (11 control and 12 stroke) whose quadriceps were electrically stimulated for twitch contractions before and after the fatiguing contraction, women had a smaller percent reduction in resting twitch amplitude than men after the fatiguing task (Figure 2, main effect sex, *p* = 0.001, η_p_^2^ = 0.447). Controls had a larger percent decline in resting twitch amplitude compared with the stroke subjects (Figure 2, main effect *p* = 0.006, η_p_^2^ = 0.336). There was no interaction effect of sex × group (*p* = 0.59).

VM EMG increased during fatiguing contractions for all groups similarly (men control 26% ± 57%, men stroke 32% ± 25%, women control 27% ± 15%, women stroke 24% ± 21%). There were no main effects for sex (*p* = 0.06) or group (*p* = 0.14) and no interaction effects (*p* = 0.27).

**Figure 3:**
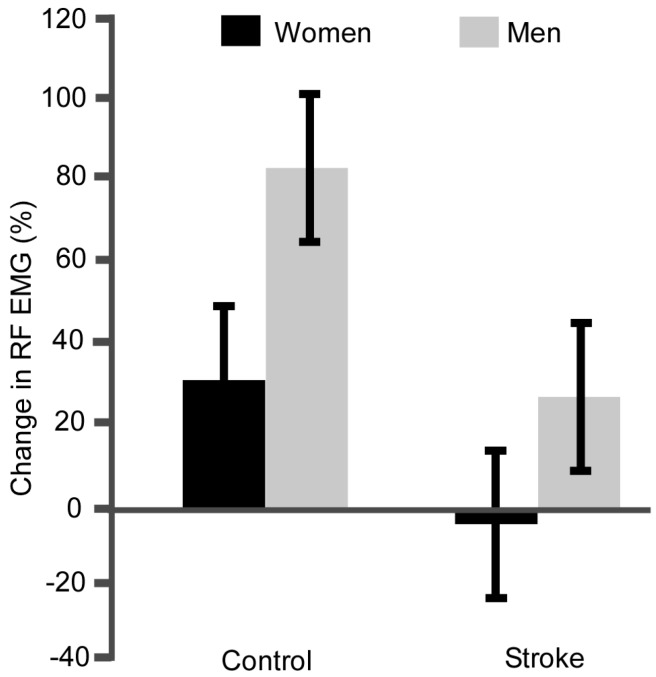
Percent change in Rectus Femoris EMG. On average, people with stroke had less change in RF EMG than controls (*p* < 0.01) and men had a larger percent increase in RF compared with women (*p* < 0.01). Women with stroke had the least change in RF EMG magnitude compared with all other groups (sex effect, *p* ≤ 0.01).

Controls had a larger percent increase in RF EMG compared with the stroke participants (Figure 3, group effect, *p* < 0.01, η_p_^2^ = 0.33) and men had a larger percent increase in RF compared with women (Figure 3, sex effect, *p* < 0.01, η_p_^2^ = 0.278). The percent change in RF EMG of the women with stroke was less than all other groups (Figure 3, *p* ≤ 0.01).

### 3.3. Relationship between Neuromuscular Fatigue Metrics and Clinical Measures

Task duration was not correlated with the Fugl–Meyer Scores, Berg Balance Scores or self-selected walking speeds (all *r*^2^ ≤ 0.09, *p* ≥ 0.16). The Berg Balance Score was correlated with the percent change in resting twitch amplitude (*r*^2^ = 0.36). Individuals with larger declines in twitch amplitude had higher Berg Balance Scores. Self-selected walking speed was positively correlated with post fatigue contraction rates (*r*^2^ = 0.35, *p* = 0.017), pre- (*r*^2^ = 0.36, *p* = 0.014) and post-relaxation rates (*r*^2^ = 0.5, *p* = 0.002), and baseline resting twitch amplitudes (*r*^2^ = 0.42, *p* = 0.021).

## 4. Discussion

The primary novel finding of the study was that the sex differences in fatigability for an intermittent isometric contraction of the knee extensors persist in people with stroke. Furthermore, data from the study suggest that mechanisms for the sex difference are similar to that of healthy controls. This is supported by the following findings: (1) as observed in the control group, the men with stroke had a shorter time to failure than the women with stroke, although the individuals with stroke had a shorter task duration than controls; (2) the slowing of relaxation rates (from MVCs) was the least for women with stroke compared with the other groups; (3) the resting twitch declined for all groups but more so for the men than the women across both the control and stroke groups, and more for the controls than the people with stroke; and (4) EMG of the vastus medialis and rectus femoris increased in all groups throughout the fatiguing protocol with the exception that women with stroke showed minimal change in the EMG activity of the rectus femoris. Although people with stroke are more fatigable than healthy controls, the women are less fatigable than the men across both groups. These results indicate that women with stroke have less fatigue within the muscle (peripheral fatigue) than men with stroke due to altered neural strategies in muscle activation during fatiguing contractions.

### 4.1. Sex Differences in Baseline Strength Persist with Stroke

The knee extensor muscles of individuals with stroke were weaker than the controls, and women were weaker than the men for both controls and people with stroke. The relative sex differences in strength for the control and stroke groups were 46% and 49%, respectively. This is consistent with findings in nonstroke control populations, showing that the limb muscles of men are usually stronger than women [5] and showing that sex differences in strength persist with stroke. In healthy individuals, differences in strength between people and groups is highly correlated with muscle mass [28] with minimal differences between men and women in their ability to activate the available muscle [24,29]. Following stroke, impaired neural activation of the muscle by the cortex [15,30,31] plays a larger role in decrements in strength than for healthy controls. Coupled with muscle atrophy, both neural and muscular mechanisms contribute to sarcopenia [32] and the associated weakness. Our results show that while people with stroke are weaker than healthy controls, the sex differences in baseline strength post-stroke are similar and that the contribution of the neural and muscular mechanisms for the reduction in strength with stroke are probably similar for the sexes. This is corroborated by our data indicating a main of effect of sex but not group for the amplitude of the resting twitch at baseline.

### 4.2. Sex Differences in Time to Task Failure Maintained with Stroke

People with stroke were more fatigable than healthy controls for the knee extensor muscles and similar with previous findings in the paretic hip flexors during sustained and maximal dynamic contractions [6,7]. Relative declines in MVCs did not differ between stroke and control groups, indicating that at task failure all groups were at similar levels of fatigability and that motivation was likely similar. Our study also suggests the mechanisms differed across the groups and the sexes. The larger percent declines in resting twitch by the controls (Figure 2) is consistent with other studies that suggest that central (neural) factors usually contribute more to fatigability post-stroke [15] than healthy controls. This is in contrast to healthy controls in which peripheral factors are a major contributor to fatigability [33], although the neural contribution can be up to 25% of force reductions during a fatiguing contraction [34].

The sex differences in fatigability observed in healthy controls (men being more fatigable than women) was also apparent in individuals with stroke. Thus, men with stroke had a shorter task duration compared with the men controls and women with stroke (Figure 1). The magnitude of baseline strength was not a determining factor of task duration because the men with stroke had similar baseline strength as the women controls, yet they had a shorter task duration. Furthermore, baseline strength of the men with stroke was not correlated with task duration. Finally, degree of motor impairment was not significantly different between men and women with stroke and likely did not play a primary factor in sex differences in fatigability.

### 4.3. Differences in Resting Twitch Amplitude Provides Insight into Mechanisms of Fatigue

The sex difference in fatigability for both the healthy controls and individuals with stroke was likely explained by peripheral mechanisms. Similar to the sex differences in the reduction in twitch amplitude in controls, the men with stroke had a larger relative decline in resting twitch amplitudes than the women with stroke. This indicates that the women with stroke have less peripheral fatigue compared with the men with stroke, albeit on a par with the controls.

The stroke subjects (both men and women) however, had less change in the twitch amplitude than healthy control men and women. Thus, although the stroke participants were more fatigable than healthy controls, the mechanisms for greater fatigability of the stroke subjects was less due to the fatigability of the muscles (less change in twitch amplitude) and thus was likely due to neural mechanisms.

### 4.4. Neural Strategies of Muscle Activation Differ with Stroke and Sex

Women also differed in patterns of knee extensor EMG activity (muscle activation) during the fatiguing tasks. Similar to men in both groups and women controls, women with stroke increased activation of VM during the fatiguing task. This finding is consistent with other studies showing increases in EMG magnitude as the active fibers progressively fatigue and additional motor units are recruited to compensate and sustain the force during sub-maximal fatiguing contractions [35,36]. However, unlike the other groups, women with stroke did not show an increase in activation of the RF muscle. This is similar to a lack of modulation of RF in response to a hip flexion fatigue protocol—albeit this protocol contained both men and women [9]. In healthy controls, the increase in EMG activity can be less for women than men in arm muscles [37]. Thus, one possibility is the women in each group had reduced fatigue in the muscle compared with the men, and a reduced afferent feedback (group III and IV) to the central nervous system, and less of a need for the central nervous system to recruit additional motor units. More mechanistic studies are needed to confirm why activation of the motor neuron pool in people with stroke differs between men and women.

### 4.5. Fatigue Metrics Associated with Neuromuscular Properties Relate to Walking Function

The task duration was not related to clinical measures of function reported in this study and probably reflects that the clinical measures chosen do not directly measure motor endurance (e.g., like the 6 min walk test [10]). Future studies will explore the relationship between clinical metrics of motor or functional endurance and fatiguing sub-maximal contraction protocols. We did, however, find correlations between self-selected walking speed and measures of peak contraction and relaxation rates pre- and post-fatigue. Individuals who could contract and relax their muscles faster also could walk faster. Damage to the motor cortex following stroke not only interferes with the ability to fully activate the muscle [31,30], but also likely impairs the speed with which muscles are fully activated due to stroke-related changes in rate modulation and recruitment. In addition, there was positive correlation between baseline resting twitch amplitudes and walking speed. This is likely due to muscle mass and strength.

### 4.6. Next Steps: Perceived Fatigue and Motor Performance Post-Stroke

In this study, we examined sex differences in neuromuscular fatigue which is defined as the acute exercise-induced reduction in force [8]. Here, women with stroke had less measurable peripheral fatigue (less of a reduction in resting twitch amplitude, Figure 3) and were weaker than men with stroke. This suggests that women with stroke may have impairments in the ability to neurally activate paretic muscles as compared to men with stroke which may impact motor performance. Neuromuscular fatigability is believed to contribute to impairments in the performance of the six-minute walk test post-stroke, by affecting the quality of walking and distance walked [10,14]. In addition, fatigue of the hip flexors has been shown to have larger effects on walking kinematics in those with stroke compared to healthy controls [9].

Motor performance can also be limited by “perceived” or “self-reported” fatigue in healthy [38] and patient populations such as stroke [39,40]. Although not fully explored, studies have reported that women report more perceived fatigue than men [40,41]. We did not measure differences in the perceptions of fatigue or effort in any of the women or men in the study, but sex differences in perceived fatigue could have contributed to the differences in task duration shown here between the men and women with and without stroke. Future studies will examine the contribution of both measured motor fatigue and perceived fatigue on human performance in men and women post-stroke.

## 5. Conclusions

In summary, the data from this study suggest that sex differences in fatigability persist in people with stroke and similar to controls may involve a more fatigue resistant muscles in the women compared with the men due to fiber type and metabolic differences during contraction [22,37]. However, people with stroke also have decreased neural drive which will exacerbate the fatigability in both men and women. These results have several implications for strength training and motor recovery post-stroke. It is well documented that the degree of lower limb paresis is positively correlated with walking function [2,3]. Thus, strength training is an important component of stroke rehabilitation. In order for neural and muscular strength adaptations to occur, the muscle must be adequately activated and overloaded—followed by a period of recovery. Here, we show that women with stroke may not be activating all of the quadriceps muscles as well as the men with stroke during a fatiguing contraction which may compromise the efficacy of strength training. Future studies will investigate how to optimize adequate muscle activation in women post stroke to enhance strength and subsequent functional gains.

## Figures and Tables

**Table 1:** There was not a statistically significant difference in function between the women and men stroke subjects (*p* ≥ 0.4 for Lower Extremity Fugl–Meyer, Self-Selected Walking Speed, and Berg Balance Test).

Subject	Sex	Age (Years)	Time since Stroke (Months)	Lower Extremity Fugl–Meyer	Self-Selected Walking Speed (m/s)	Berg Balance Test
S2	women	66	93	17	0.28	38
S3	women	62	270	30	1.22	47
S5	women	57	235	14	0.40	46
S6	women	76	51	30	1.09	48
S9	women	79	52	27	0.66	49
S13	women	62	191	12	0.84	46
S16	women	64	110	32	0.80	54
S17	women	80	94	21	0.60	26
Average (±SD)	women	68.25 ± 9	137.00 ± 84	22.88 ± 7.90	0.74 ± 0.3	44.25 ± 9
S1	men	55	118	29	0.96	45
S4	men	48	439	23	1.35	55
S7	men	67	17	11	0.14	24
S8	men	48	140	21	0.34	43
S10	men	55	65	22	0.83	42
S11	men	67	113	19	0.67	48
S12	men	64	65	27	1.24	49
S14	men	47	49	28	1.04	52
S15	men	59	110	24	1.10	49
S18	men	62	90	21	0.90	39
Average (±SD)	men	57 ± 8	120.6 ± 117	22.5 ± 5	0.86 ± 0.4	44.6 ± 8
